# Meningeal lymphatic vessel dysfunction driven by CGRP signaling causes migraine-like pain in mice

**DOI:** 10.1172/JCI182556

**Published:** 2024-08-01

**Authors:** Jean-Leon Thomas, Emmanuelle A.D. Schindler, Christopher Gottschalk

**Affiliations:** 1 Department of Neurology, Yale University School of Medicine, New Haven, Connecticut, USA.; 2 Paris Brain Institute, Université Pierre et Marie Curie Paris 06 UMRS1127, Sorbonne Université, Paris, France.; 3 Neurology Service and; 4 Headache Center of Excellence, Veterans Affairs Connecticut Healthcare Sytem, West Haven, Connecticut, USA.

## Abstract

Migraines are a type of headache that occur with other neurological symptoms, but the pathophysiology remains unclear. In this issue of the *JCI*, Nelson-Maney and authors used constitutive and inducible knockouts of the CGRP receptor components, elegantly demonstrating an essential function of CGRP in modulating meningeal lymphatic vessels (MLVs) in migraine. CGRP was shown to induce rearrangement of membrane-bound gap junction proteins in MLVs, resulting in a reduced CSF flux into cervical lymph nodes. The authors also provided evidence of a primary role for CGRP in modulating neuro-immune function. Finally, by showing that blocking CGRP signaling in MLVs attenuated pain behavior associated with acute migraine in rodents, the authors provided a target for pharmacological blockade of CGRP in relation to primary headache disorders.

## Migraine and the rational for targeting CGRP

Migraine is a disorder characterized by recurring headache attacks accompanied by other neurological symptoms and affects 15% of the world’s population. The frequency and severity of these attacks have substantial impacts on socioprofessional and interpersonal function, and migraine ranks second or third worldwide as a cause of disability. Despite the availability of a variety of therapies targeting numerous neurotransmitter and other biological systems, many patients remain refractory to these treatments. The development of new and efficient methods to manage migraine is therefore needed and will have substantial impact on global health. In this issue of the *JCI*, a thoughtful study conducted by Nelson-Maney et al. (1) calls attention to lymphatic vasculature of the meninges as a potential therapeutic antimigraine target, via calcitonin gene–related peptide (CGRP) signaling, which mediated multiple effects on pain, neuroinflammation, and cerebrospinal fluid (CSF) efflux in a model of migraine attack.

In clinical practice, triptans, which are synthetic serotonin receptor agonists, are first-line agents for the acute treatment of migraine, although these drugs may not be effective or may be contraindicated for certain patients. Likewise, preventive migraine agents, such as beta blockers, tricyclic antidepressants, antiepileptics, or botulinum toxin, may be ineffective or contraindicated. Several medications targeting the CGRP signaling axis have been developed and recently approved by the U.S. FDA.

The rationale for targeting CGRP for migraine stems from decades of evidence demonstrating a role for the proinflammatory, vasodilatory peptide in this disorder. CGRP is elevated during acute migraine attacks (2, 3) and between attacks in people with migraines compared with people in control groups (4, 5). Peripheral CGRP levels are also elevated in individuals with migraines who respond to acute and preventive therapies (2–4) and then decrease after drug treatment. Given that CGRP release correlated with migraine and cluster headache attacks in vivo and that CGRP infusion could precipitate attacks in humans susceptible to either headache type, pharmaceutical trials have been conducted to block CGRP activity. After nearly thirty years of clinical study, the blockade of CGRP using monoclonal antibody technology has proven successful to reduce migraine attacks, as have small molecule receptor antagonists (such as gepants) for termination of acute attacks. Despite these successes, the primary function of CGRP in the CNS has remained obscure. CGRP contributes to vasodilation in response to ischemia, but whether it also participates into other homeostatic brain functions remains unclear. The precise location of the targets of CGRP blocking monoclonal antibodies, which cannot access the brain tissue from the blood circulation and must thus reside outside the blood brain barrier, also remains unknown.

**Figure 1:**
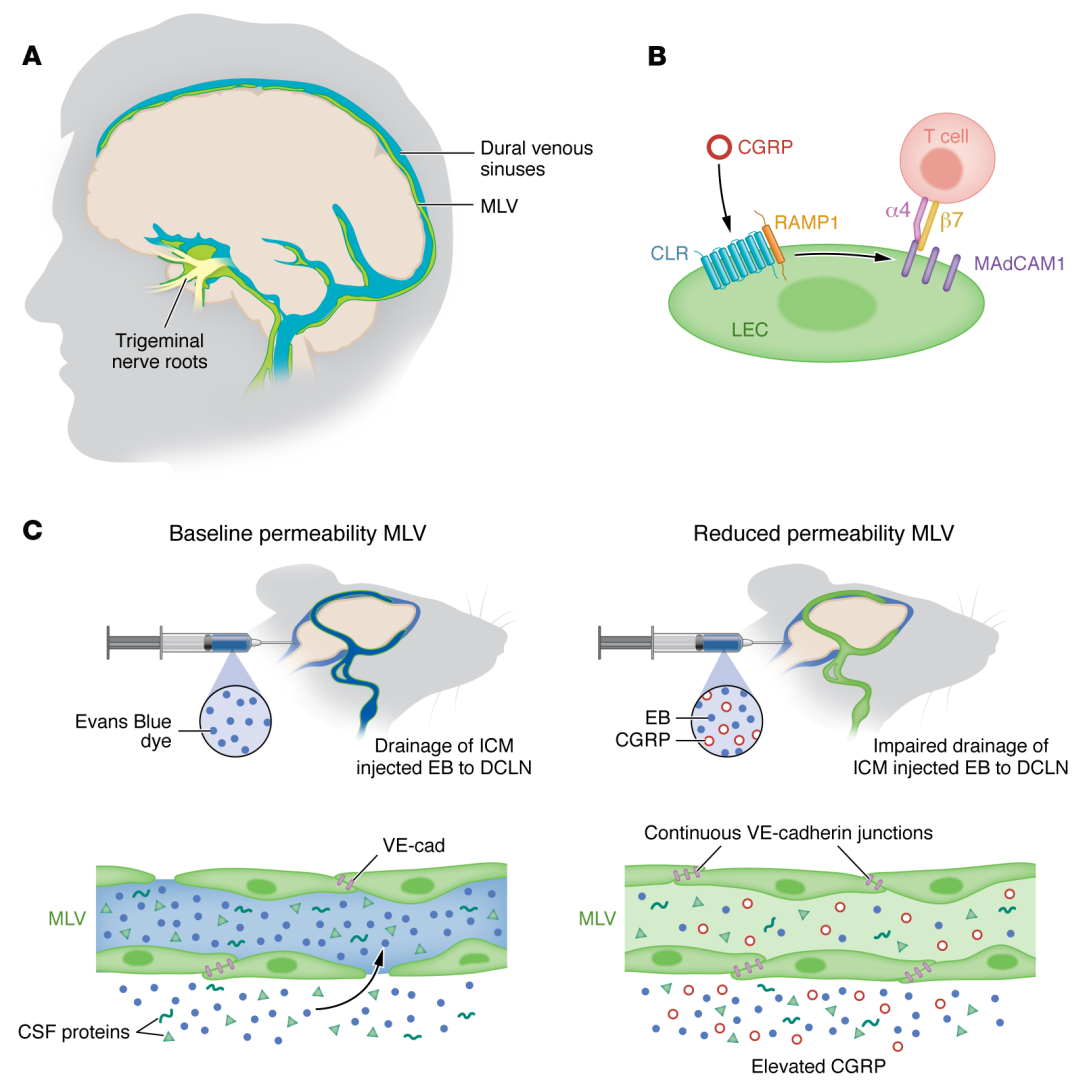
CGRP signaling mediates the functional interplay between MLVs and migraine. (**A**) CGRP-expressing trigeminal nerve roots are in close proximity to CGRP receptor–expressing MLVs. (**B**) In deep cervical lymph nodes (DCLNs), MAdCAM1-expressing lymphatic endothelial cells (LECs) interact with CD4^+^ T cells to promote pathologic lymphatic vessel remodeling via CGRP signaling. (**C**) Decreased permeability in MLVs reduces CSF outflow to DCLNs.

Nelson-Maney and colleagues focused their study on CGRP signaling in MLVs based on a set of strong premises regarding CGRP receptor expression and function in peripheral lymphatics (1). The CGRP receptor complex is highly expressed in lymphatic endothelial cells compared with blood endothelial cells (6), and CGRP receptor signaling is required during lymphatic development and maintenance in the adult. Homozygous loss-of-function mutations of the CGRP receptor in humans and mice are associated with nonimmune hydrops fetalis (7, 8). CGRP is also known to target the smooth muscle and endothelial cells of large cerebral blood vessels of the meninges (9). In particular, the release of CGRP during a migraine attack occurs from trigeminal C-fibers and causes vasodilation of dural venous sinuses that are in contact with dural MLVs (10, 11) (Figure 1). These MLVs connect the central and peripheral immune systems and provide the brain with a drainage system for the efflux of CSF and waste generated by neural cell activity (12). Therefore, expression and functional evidence supports the hypothesis that CGRP/CGRP receptor signaling may act in MLVs to regulate brain fluid drainage and neuroimmune inflammation during migraine.

## Migraine models and pathophysiological findings

Nelson-Maney and authors conducted their studies in mouse models with either conditional deletion of the CGRP receptor in lymphatic endothelial cells (*Calcrl ^fl/fl^; Vegfr3^CreERT2/+^* or *Calcrl ^iLEC^* mice) or constitutive loss of function of the requisite allosteric modulator Ramp1 (*Ramp1^–/–^* mice). To faithfully recapitulate aspects of human migraine, the authors used nitroglycerin (NTG), a potent nitric oxide donor and a vasodilator that sensitizes the trigeminovascular system and causes release of CGRP. NTG was administered intraperitoneally, resulting in migraine-like pain and associated behaviors that were measured in animals using two independent behavioral metrics—facial grimace and light avoidance/movement. CGRP was also injected intrathecally to directly stimulate the MLVs (1).

After NTG injection, *Calcrl^iLEC^* and *Ramp1^-/-^* mutants showed no changes in the morphology of the MLV vasculature, but the manifestations of pain were reduced in both genetic models compared with controls. NTG thus induced CLR/RAMP1–dependent migraine-like responses. This study directly demonstrates that lymphatic CGRP signaling is a major player in the pathophysiology of migraine pain (1).

The authors next investigated the transcriptional changes evoked by NTG in MLVs in vivo using the established model of *Rpl22^HA/+^; Lyve1^Cre^* mice, which enables the collection of actively translating mRNAs from Lyve1-Cre^+^ MLV endothelial cells. Microarray analysis of *Lyve1*-*Cre^+^* ribosome-associated mRNAs revealed a gene signature indicative of an immune response within the meninges following NTG treatment, including several Th1 and Th2 activation pathways and established serum biomarkers of migraine such as C-related peptide (CRP) and pentraxin3 (PTX3), which supports the model of neuro-vascular-immune crosstalk during migraine responses. Cultures of human lymphatic endothelial cells exposed to CGRP peptides confirmed the upregulation of markers of lymphatic vascular-immune interactions, including connexin 47, MAdCAM1, and pentraxin3, previously identified by transcriptomic analysis, as well as their regulation by CGRP signaling (1).

MAdCAM1 is a potent adhesion molecule for α4/β7 integrin–positive (LPAM_1-positive) T cells, while pentraxin3 expression characterizes capillary lymphatic endothelial cells that interact with immune cells and promote pathologic lymphatic vessel remodeling. One of the most exciting findings of Nelson-Maney et al. (1) concerns the interaction between these molecules in migraine pain. Immunolocalization of pentraxin3 on dissected meninges revealed focal expression at the endpoints of MLV capillaries, which increased upon NTG treatment. In this regard, multispectral flow cytometry analysis of cervical lymph nodes showed that the population of LPAM1^+^ CD4^+^ T cells was also increased by NTG treatment. These observations raised the attractive proposition that MLV capillary endpoints are immunologically primed by NTG-induced CGRP signaling. NTG-induced priming of MLVs yielded increased expression of MAdCAM1 and pentraxin3 (1). This altered expression may then facilitate the binding and egress of meningeal LPAM1^+^CD4^+^ T cells into draining cervical lymph nodes. However, the precise role of these cells in migraine is currently unknown.

Nelson-Maney and authors completed their exploration by investigating how CGRP alters lymphatic uptake and drainage of CSF through the cytological remodeling of lymphatic endothelial cells and interendothelial cell junctions. In cultured human lymphatic endothelial cells, CGRP was found to induce formation of continuous, nonpermeable VE-cadherin junctions, involving activation of ERK-CREB phosphorylation. In vivo, intrathecal injection of Evans Blue was utilized as an innovative method to quantify CSF uptake by MLVs and CSF drainage to the deep cervical lymph nodes in CGRP receptor mutants. In a final tour de force, the authors included CGRP in the Evans Blue injection, causing reduction of dye uptake by MLVs in *Calcrl^fl/fl^* mice while *Calcrl^iLEC^* CGRP receptor mutants were unresponsive. CGRP signaling was thus demonstrated to be required for VE-cadherin linear junction formation at MLV endpoints to render MLVs impermeable and to impair their drainage function (1).

## Discussion

Collectively, Nelson-Maney et al. (1) break new ground, revealing that MLVs are key mediators of NO- and CGRP-induced migraine pain and that CGRP signaling simultaneously promotes immune cell egress and alters fluid uptake into MLVs.

Until now, debate has raged concerning the central or peripheral origin of migraine attacks. The present study outlines a most detailed window on the location of a crucial CGRP-mediated effect: the MLVs located in the dura mater outside of the CNS (1). Further research will clarify whether CGRP antagonists act directly at the interface between MLVs and trigeminal nerve terminals.

The impaired fluid uptake induced by CGRP in MLVs compromises CSF drainage to cervical lymph nodes, but it may also alter the clearance of brain waste by the glymphatic system. MLVs function downstream of the glymphatic system (13), collecting brain-derived solutes and antigens at the level of dural perisinusal spaces (14–16). Although Nelson-Maney and colleagues have not investigated the consequences of CGRP treatment on CSF drainage of brain parenchyma in their mouse genetic models (1), previous studies have reported shrinkage of the paravascular space surrounding penetrating arteries and pial vessels (17), possibly via astrocytic endfeet swelling (9, 18). Impaired brain waste clearance indirectly caused by CGRP signaling may thus provide an alternative mechanism to explain the intellectual impairment often reported during acute migraine attacks, particularly deficits in attention and memory (19).

Magnetic resonance imaging of meningeal lymphatic drainage and CSF parenchymal drainage (LG-MRI) is now available in humans (20), and dysfunctions in these systems have been identified in some studies of patients with migraines (21, 22). Future investigations of the effects of CGRP-targeted treatments on these imaging measures, especially at the level of the trigeminal nerve roots that are adjacent to a dense network of MLVs (16), will surely help to further characterize the relationship between dural lymphatics and CSF parenchymal drainage in migraine (Figure 1). LG-MRI could provide a prognostic tool by quantifying the relationship of chronically impaired MLV drainage with elevated interictal CGRP serum levels or the severity of chronic migraine impairment. LG-MRI could also be used as a survey tool to assess the benefit of CGRP blockade on CSF drainage. Finally, the present finding provides a tantalizing suggestion that constitutive activation of CGRP-induced alterations in MLV function may be a key alteration in other headache disorders, such as persistent post-traumatic headache (PPTH), which is clinically indistinguishable from chronic migraine and the most common long-term consequence of mild traumatic brain injury.

## Version 1 08/01/2024

Electronic publication
